# MNT inhibits lung adenocarcinoma ferroptosis and chemosensitivity by suppressing SAT1

**DOI:** 10.1038/s42003-024-06373-5

**Published:** 2024-06-03

**Authors:** Guangyin Zhao, Jiaqi Liang, Yuchen Zhang, Guangyao Shan, Yunyi Bian, Jie Gu, Cheng Zhan, Di Ge

**Affiliations:** Department of Thoracic Surgery, Zhongshan Hospital, Fudan University, Shanghai, China

**Keywords:** Non-small-cell lung cancer, Cell death

## Abstract

Ferroptosis, a type of iron-dependent non-apoptotic cell death, plays a vital role in both tumor proliferation and resistance to chemotherapy. Here, our study demonstrates that MAX’s Next Tango (MNT), by involving itself in the spermidine/spermine N1-acetyltransferase 1 (SAT1)-related ferroptosis pathway, promotes the proliferation of lung adenocarcinoma (LUAD) cells and diminishes their sensitivity to chemotherapy. Initially, an RNA-sequence screen of LUAD cells treated with ferroptosis inducers (FINs) reveals a significant increase in MNT expression, suggesting a potential link between MNT and ferroptosis. Overexpression of MNT in LUAD cells hinders changes associated with ferroptosis. Moreover, the upregulation of MNT promotes cell proliferation and suppresses chemotherapy sensitivity, while the knockdown of MNT has the opposite effect. Through the intersection of ChIP-Seq and ferroptosis-associated gene sets, and validation by qPCR and western blot, SAT1 is identified as a potential target of MNT. Subsequently, we demonstrate that MNT binds to the promoter sequence of SAT1 and suppresses its transcription by ChIP-qPCR and dual luciferase assays. Restoration of SAT1 levels antagonizes the efficacy of MNT to inhibit ferroptosis and chemosensitivity and promote cell growth in vitro as well as in vivo. In the clinical context, MNT expression is elevated in LUAD and is inversely connected with SAT1 expression. High MNT expression is also associated with poor patient survival. Our research reveals that MNT inhibits ferroptosis, and impairing chemotherapy effectiveness of LUAD.

## Abstract (web summary)

The transcription factor MNT binds to the SAT1 promoter region and represses its transcription, leading to ferroptosis and chemotherapy resistance in lung adenocarcinoma.

## Introduction

Lung adenocarcinoma (LUAD) is a malignant tumor characterized by high proliferation rates. It represents ~ 40 % of all cases of lung cancer and is the predominant pathological subtype associated with a globally unfavorable prognosis^1,2^. Despite the substantial advancements in LUAD therapy throughout the last couple of decades, chemotherapy continues to be an essential therapeutic approach^3–5^. Nevertheless, the development of chemoresistance poses a significant challenge in enhancing the efficacy of therapy for LUAD. Hence, it is imperative to suppress the proliferation capacity and enhance the chemosensitivity in the treatment of LUAD.

Initially described in 2012, Ferroptosis represents a newly identified form of programmed death of cells that relies on the presence of iron ions^6,7^. A close relationship between ferroptosis and tumorigenesis has been established, as the lack of ferroptosis can stimulate tumorigenesis and enhance tumor proliferation.^8,9^. Significantly, the induction of ferroptosis has the potential to enhance chemotherapy sensitivity in the context of LUAD treatment^6^.

SAT1, also known as spermidine/spermine N1-acetyltransferase 1, serves as the principal enzyme that regulates the rate of the polyamine metabolic pathway, which is crucial for the proliferation and viability of eukaryotic cells.^10,11^. SAT1 is crucial in responding to multiple stress responses, including oxidative stress, heat shock, and inflammatory stimuli. More importantly, aberrantly expressed SAT1 is closely related to cancer development, including LUAD^10,12^. Recently, SAT1 was reported to be associated with the regulating process of ferroptosis in tumor cells^11,13^. However, the mechanism by which cancer regulates SAT1 to affect ferroptosis is still unidentified.

Transcription factor MNT (MAX’s Next Tango) belongs to the MXD family, playing an essential role in cell proliferation, differentiation, and cellular transformation, and abnormal expression of MNT is strongly associated with cancer^14^. Here, MNT was discovered to be elevated in LUAD cells following the administration of FINs, suggesting that MNT is potentially connected with regulating the ferroptosis of LUAD. Nevertheless, MNT’s role in ferroptosis and its mechanism, however, is yet to be established.

Hence, we determined MNT as a translational suppressor of SAT1 and demonstrated that up-regulating MNT hinders the ferroptosis occurrence, consequently leading to a suppression of chemotherapy sensitivity while promoting the proliferation of LUAD cells.

## Results

### MNT involves ferroptosis in LUAD

**Fig. 1:**
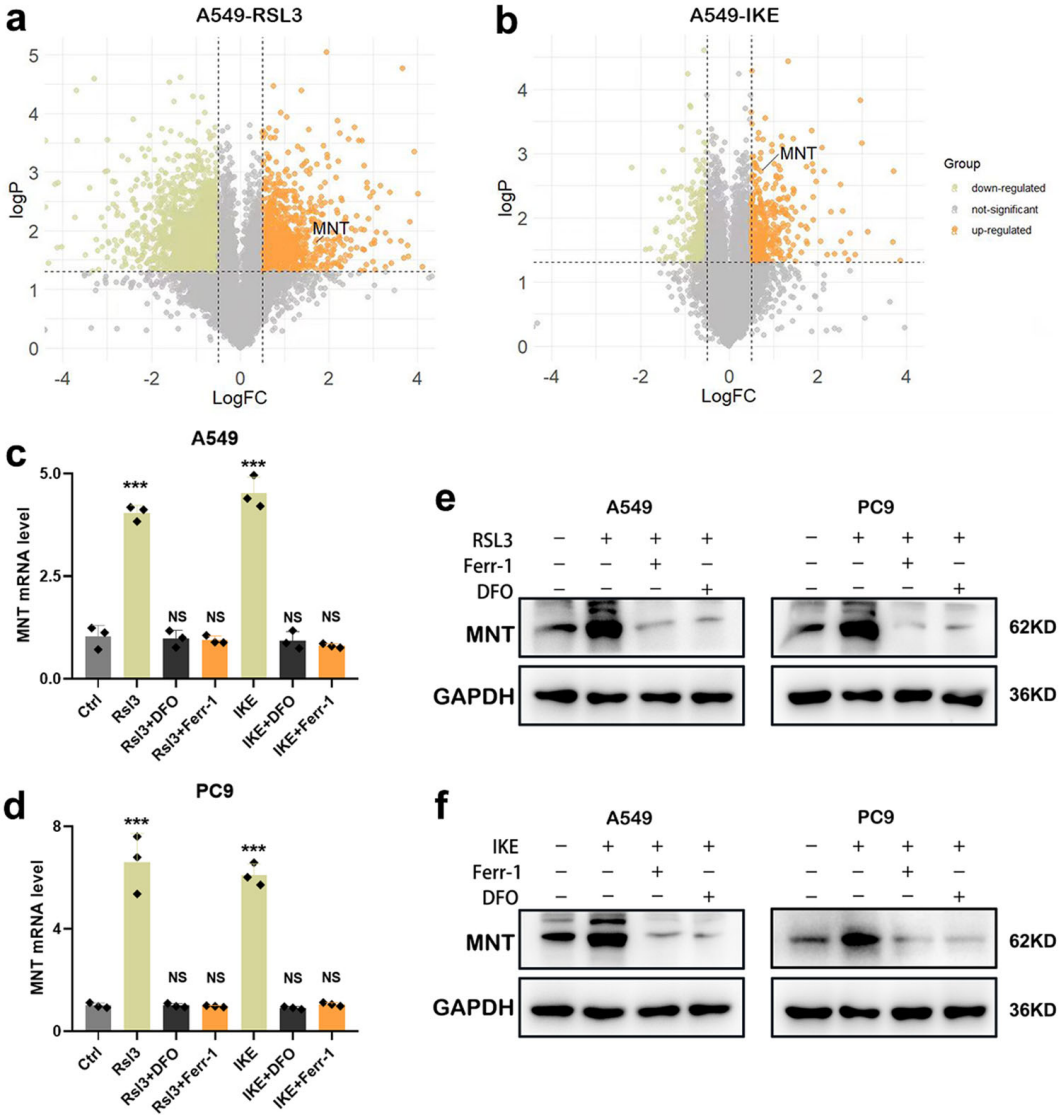
Identification of MNT involved in LUAD ferroptosis. **a**, **b** Volcano plot exhibiting MNT involved in regulating ferroptosis induced by RSL3 and IKE (2.5 μM RSL3 and 8 μM IKE for 5 h). **c**, **d** MNT RNA levels in A549 and PC9 cell lines were assessed following treatment with RSL3/IKE (2.5 μM RSL3 and 8 μM IKE for 5 h), DFO + RSL3/IKE, or Ferr-1 + RSL3/IKE. **e**, **f** MNT protein levels were evaluated in A549 and PC9 cell lines after treatment with RSL3/IKE (2.5 μM RSL3 and 8 μM IKE for 5 h), DFO + RSL3/IKE, or Ferr-1 + RSL3/IKE. Data were analyzed by Student’s *t*-test and presented by mean ± SD in triplicate. ns, not significant; *p < 0.05; **p < 0.01; ***p < 0.001.

We first treated A549 cells with RSL3, IKE (imidazole ketone Erastin),and DMSO respectively, and then performed RNA-seq on the treated and non-treated groups. The results reveal that MNT was markedly elevated in the groups treated with RSL3 and IKE in comparison to the group treated with DMSO (Fig. 1a, b). To validate this finding, we further examined the changes in MNT mRNA and protein levels under different treatments of LUAD cells using qPCR and western blot. Consistently, the RNA and protein levels of MNT were significantly upregulated after treatment with FINs in both A549 and PC9 cells (Fig. 1c–f). Intriguingly, when cells from both FINs-treated groups were concurrently exposed to a ferroptosis inhibitor, either Ferrostatin-1 (Ferr-1) or Deferoxamine (DFO), we observed no significant difference in MNT expression levels compared to the control group. (Fig. 1c–f). The above results highly indicate the involvement of MNT in the ferroptosis regulation of LUAD.

### MNT suppresses ferroptosis, enhances proliferation, and modulates chemotherapy sensitivity in LUAD cells

**Fig. 2:**
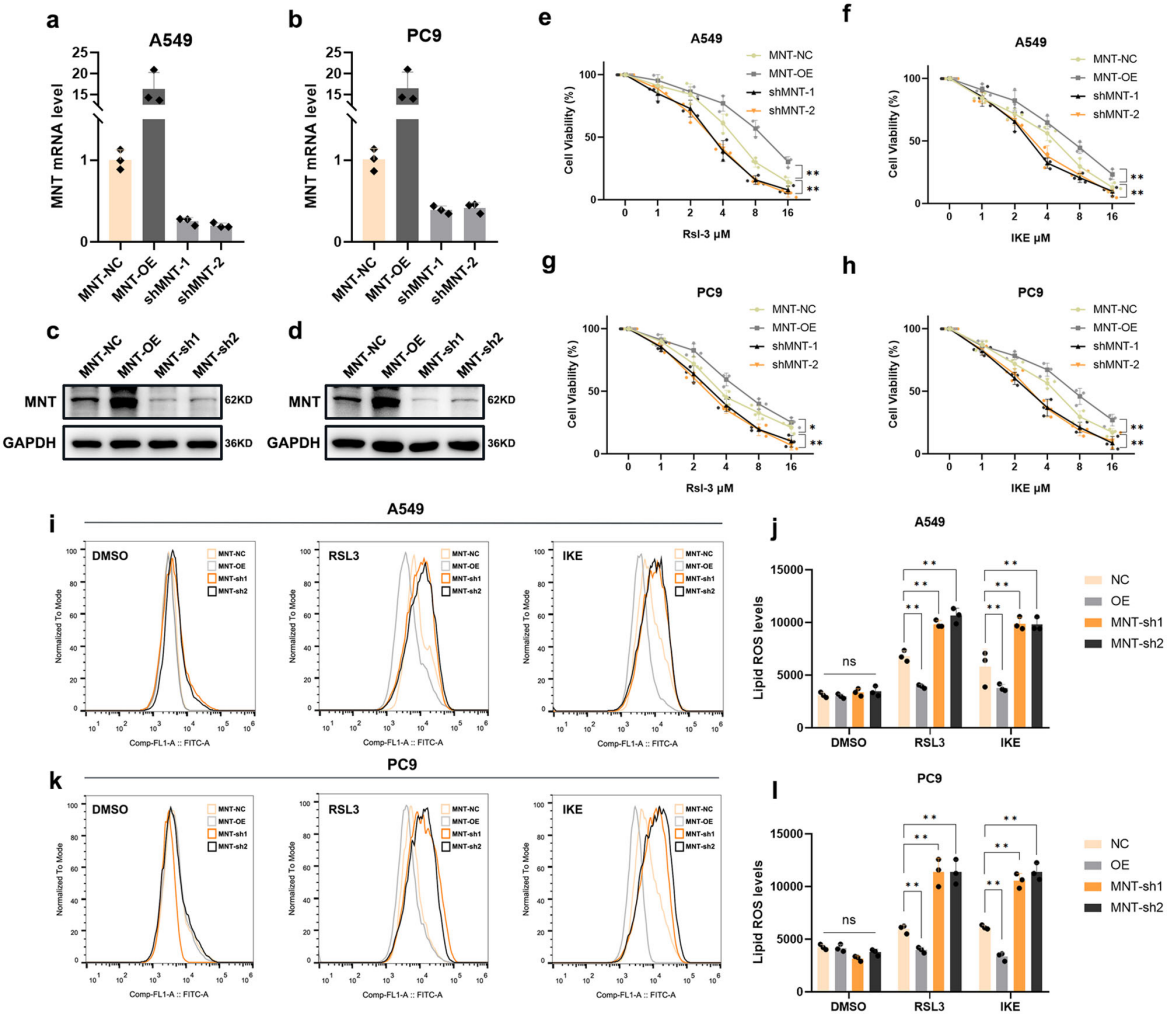
RSL3 and IKE-induced cytotoxicity and intracellular ROS levels in LUAD cell lines are impacted by dysregulated MNT expression. **a**–**d** MNT overexpression and knock-down in A549 and PC9 cells were verified by qRT-PCR and western blot. **e**–**h** After 24 h of treatment with various dosages of RSL3 and IKE, A549 and PC9 cells with various degrees of MNT expression were detected using the CCK-8 assay. **i**–**l** Lipid peroxidation was measured by flow cytometry after C11-BODIPY staining in MNT-NC, MNT-OE, and MNT-sh treatment by RSL3 or IKE (2.5 μM RSL3 and 8 μM IKE for 5 h). MNT-NC, MNT-Negative Control; MNT-OE, MNT-Overexpression; MNT-sh1, MNT- Short Hairpin RNA1; MNT-sh2, MNT- Short Hairpin RNA2. Data were analyzed by Student’s *t*-test or two-way ANOVA and presented by mean ± SD in triplicate. ns, not significant; *p < 0.05; **p < 0.01; ***p < 0.001.

We next created stable cell lines (A549 and PC9) with MNT overexpression (MNT-OE), knockdown (MNT-sh1 and sh2), and corresponding negative control (MNT-NC) via lentiviral transfection. The efficiency of transfection was measured by qPCR and western blot and showed that MNT was stably expressed or knocked down in both cell lines (Fig. 2a–d, Fold change values: A549, OE/NC 16.19, *p* = 0.022; sh1/NC 0.25, *p* = 0.004; sh2/NC 0.20, *p* = 0.005; PC9, OE/NC 16.35, *p* = 0.021; sh1/NC 0.39, *p* = 0.009; sh2/NC 0.42, *p* = 0.008). After processing with FINs (RSL3 or IKE), the MNT-OE cells had relatively higher cellular activity and significantly lower severity of lipid peroxidation inside cells in comparison to the MNT-NC group (Fig. 2e–l). Conversely, MNT-sh cells displayed a significant decrease in cellular activity alongside a pronounced enhancement in intracellular lipid peroxidation severity (Fig. 2e–). This indicates that MNT expression may play a role in preventing ferroptosis in LUAD cells.

**Fig. 3:**
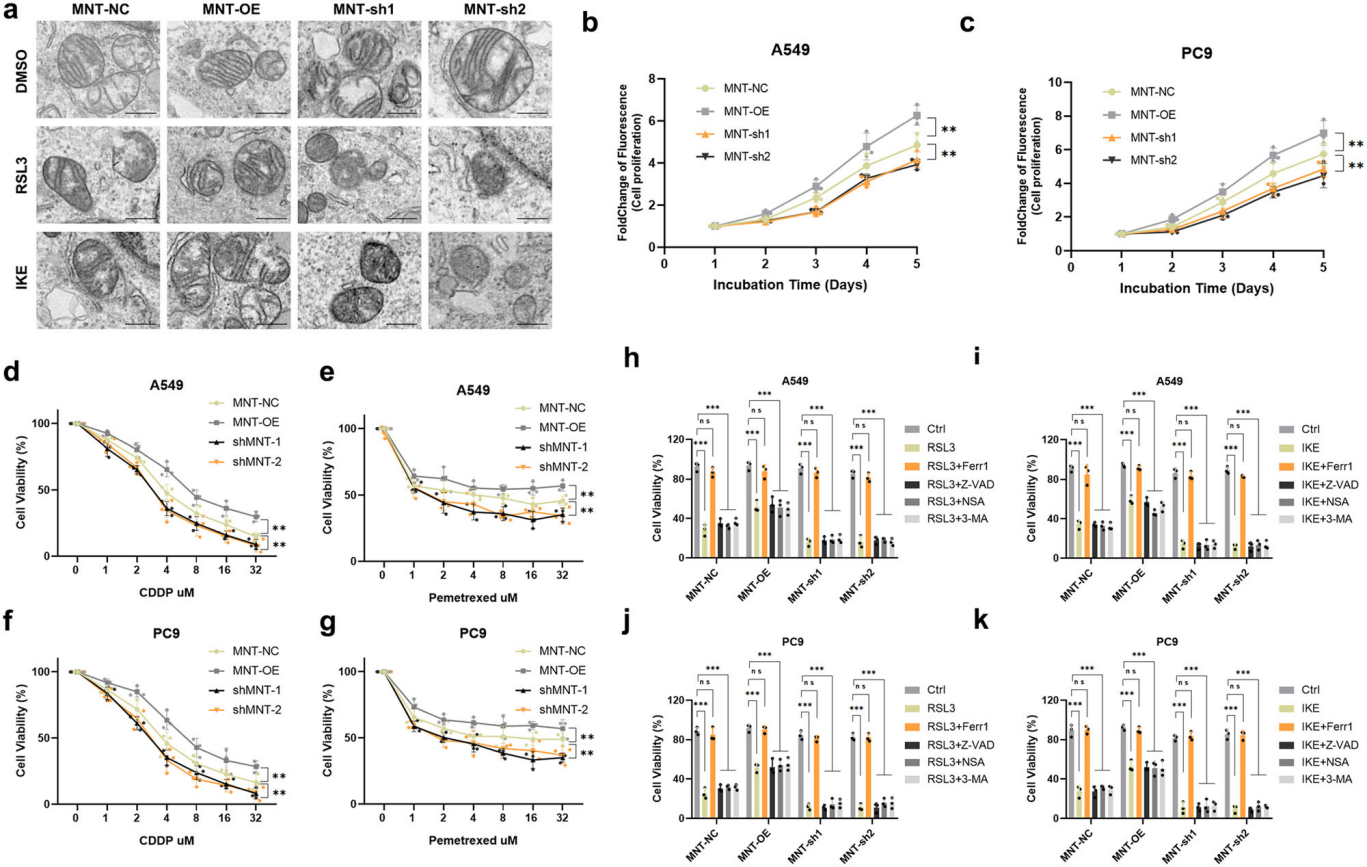
In LUAD cells treated with RSL3 or IKE, dysregulation of MNT expression results in aberrant mitochondrial shape and impairs the cells’ capacity of proliferation and susceptibility to chemotherapy. **a** Characteristic alterations of mitochondria in A549 cells treated with RSL3 or IKE (2.5 μM RSL3 and 8 μM IKE for 5 h) analyzed by TEM, Scale bars: 50 nm. **b**, **c** The capacity of A549 and PC9 cells to proliferate is impacted by MNT expression dysregulation. **d**–**g** Dose-toxicity curves showing the viability of A549 and PC9 cells transfected with MNT-NC, MNT-OE, and MNT-sh upon CDDP or Pemetrexed treatment at the indicated concentrations for 48 h. **h**–**k** A549 and PC9 cells transfected with MNT-NC, MNT-OE, and MNT-sh were examined for vitality assessed 24 h after being treated with RSL3/IKE (2.5 μM RSL3 and 8 μM IKE for 5 h), RSL3/IKE + Ferr-1, RSL3/IKE + Z-VAD, RSL3/IKE + NSA, or RSL3/IKE + 3-MA. Data were analyzed by Student’s *t*-test or two-way ANOVA and presented by mean ± SD in triplicate. ns, not significant; *p < 0.05; **p < 0.01; ***p < 0.001.

Morphological changes in mitochondria, such as reduction in mitochondrial volume, condensed mitochondrial membrane density, reduction or disappearance of mitochondrial cristae, and rupture of the outer mitochondrial membrane are important phenotypes of cells undergoing ferroptosis^15^. Transmission electron microscopy (TEM) was next conducted to detect the effection of MNT on ferroptosis in LUAD cells. Upon FIN stimulation, MNT-sh A549 cells exhibited more shrunken mitochondria, higher membrane density, and reduced mitochondrial cristae compared to either the MNT-NC or OE groups (Fig. 3a). “Ballooning” is a phenotype manifested by cells undergoing ferroptosis, characterized by the emergence of clear, rounded morphology predominantly composed of vacuolated cytosol^16,17^. In our experiments, LUAD cells with knocked-down MNT exhibited a more pronounced ballooning phenotype upon treatment with FINs, whereas MNT-OE cells showed no significant morphological changes (Supplementary Fig. 1a). Using flow cytometry for Tetramethylrhodamine, methyl ester (TMRM) assay, we found that TMRM fluorescence significantly decreased in MNT-knockdown LUAD cells and increased in the MNT-OE group compared to the control group (Supplementary Fig. 1b, c).

To examine the effects of MNT expression on LUAD cell proliferation and chemotherapy sensitivity, we proceeded with a CCK-8 experiment. We found that the downregulation of MNT significantly impeded LUAD cell proliferation (Fig. 3b, c) while enhancing their susceptibility to chemotherapeutic drugs (CDDP and Pemetrexed) (Fig. 3d–g). However, upregulation of MNT obtained the opposite result (Fig. 3b–g). Subsequently, the promotion of ferroptosis in LUAD cells by MNT-sh can be counteracted by ferroptosis inhibitor Ferr-1, but not the apoptosis inhibitor Z-VAD-FAM, necrosis inhibitors (NSA), and autophagy inhibitors (3-MA) (Fig. 3h–k). Combining these outcomes, we preliminary conclude that MNT not only inhibits ferroptosis in LUAD cells and facilitates their proliferation, but also influences their response to chemotherapy.

### The overexpression of MNT suppresses cellular ferroptosis through the downregulation of SAT1 expression

**Fig. 4:**
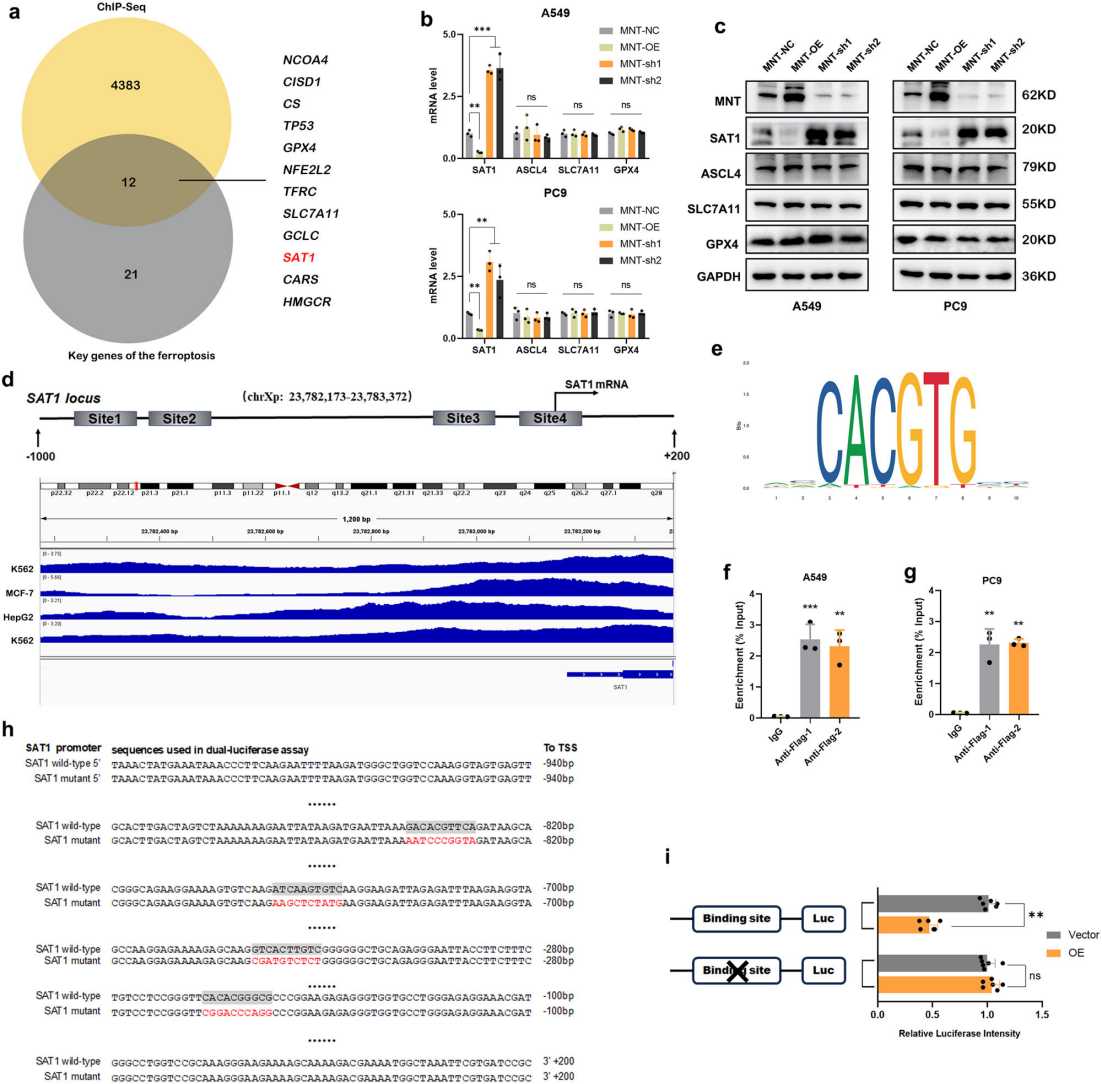
MNT regulates ferroptosis by binding to the SAT1 promoter region and repressing its transcription. **a** Venn plot showing the intersection of predicted targets of MNT, the 33 key ferroptosis genes were intersected with the results of ChIP-seq, obtaining 12 gene sets that may be target genes regulated by MNT. **b** qRT-PCR results showed differences in RNA levels of the key molecules (GPX4, SLC7A11, ASCL4 and SAT1) of ferroptosis between the MNT-NC, MNT-OE and MNT-sh of A549 and PC9 cell lines. **c** Western blot results showed differences in protein levels of GPX4, SLC7A11, ASCL4, and SAT1 of ferroptosis between the MNT-NC, MNT-OE, and MNT-sh of A549 and PC9 cell lines. **d** Genome-wide data from the ENCODE project and ChIP-seq results show the solid MNT-binding peak in the promoter region close to the TSS of SAT1. **e** Characteristic sequences predicted by JASPAR for transcriptional binding of MNT. **f**, **g** ChIP-qPCR assay depicting the enrichments of MNT binding in A549 and PC9. **h** Schematic diagram showing the possible binding sites of MNT to the critical region of the SAT1 promoter are mutated. **i** Dual luciferase activity assays to analyze the fluorescence intensity of MNT-NC and MNT-OE with or without mutations in the SAT1 promoter region. Data were analyzed by Student’s *t*-test and presented by mean ± SD in triplicate. ns, not significant; *p < 0.05; **p < 0.01; ***p < 0.001.

To explore the downstream signaling molecules of MNT inhibiting ferroptosis, we first identified MNT protein binding partners using the String database (https://cn.string-db.org/), as presented in Supplementary Fig. 2a. Subsequently, RNA-seq analysis revealed no significant differential expression among these partners. We then intersected the ChIP-Seq results of MNT in the Encode database (https://www.encodeproject.org/) with the set of key genes of the ferroptosis pathway (Fig. 4a). A total of 12 ferroptosis-related genes were screened out as potential targets of MNT. We validated these genes plus an additional star gene ACSL4, 13 genes in total, by qPCR (Fig. 4b and Supplementary Fig. 2b). The results indicated that SAT1 may be a target molecule of MNT since SAT1 was determined an important molecule that promotes ferroptosis^11^. We further validated the impact of MNT on SAT1 expression by qRT-PCR and western blot, As predicted, overexpression of MNT decreased the SAT1 level, while MNT knock-down led to the upregulation of SAT1 (Fig. 4b, c). And yet, the level of other ferroptosis key regulators, including ACSL4, SLC7A11, and GPX4 was not affected (Fig. 4b, c and Supplementary Fig. 2c, d). Additionally, we observed that knocking down MNT expression did not significantly affect SAT1 expression levels in LUAD cells treated with FINs (Supplementary Fig. 2e). These findings indicate that MNT could potentially play a role in LUAD ferroptosis by modulating the expression of SAT1.

### MNT represses SAT1 expression by interacting with its promoter region

Based on the above results, we proposed that MNT engages with the promoters of SAT1 to suppress its expression. To verify our hypothesis, we analyzed the CHIP-Seq and the genome-wide data from the ENCODE project. As anticipated, we observed a prominent MNT-binding peak in a promoter region close to the transcription start site (TSS) of SAT1 (Fig. 4d). Furthermore, the ChIP-PCR analysis also indicated notable enrichments of MNT binging in LUAD cell lines (Fig. 4f and g). These results confirmed that SAT1 is a direct transcriptional target of MNT.

To explore the functional validity of MNT binding to the SAT1 promoter, we then conducted luciferase reporter activity experiments by cloning the human SAT1 promoter sequences into the PGL4-luciferase reporter vector. To construct the negative control, the sites where MNT binds with a high possibility were predicted and modified according to the consensus motifs on the JASPAR (Fig. 4e and h). We found that MNT-OE in 293 T substantially decreased the activity of the luciferase reporter containing the wild-type (WT) SAT1 promoter sequences. However, the reporter with mutant (MU) SAT1 promoter constructs displayed no luciferase decrease in response to MNT overexpression (Fig. 4i). These results elucidated the process by which MNT firmly binds to the SAT1 promoter, thereby inhibiting its transcription.

### The expression of SAT1 counteracts the effects of MNT on ferroptosis inhibition, promotion of proliferation, and reduction of chemotherapeutic sensitivity

**Fig. 5:**
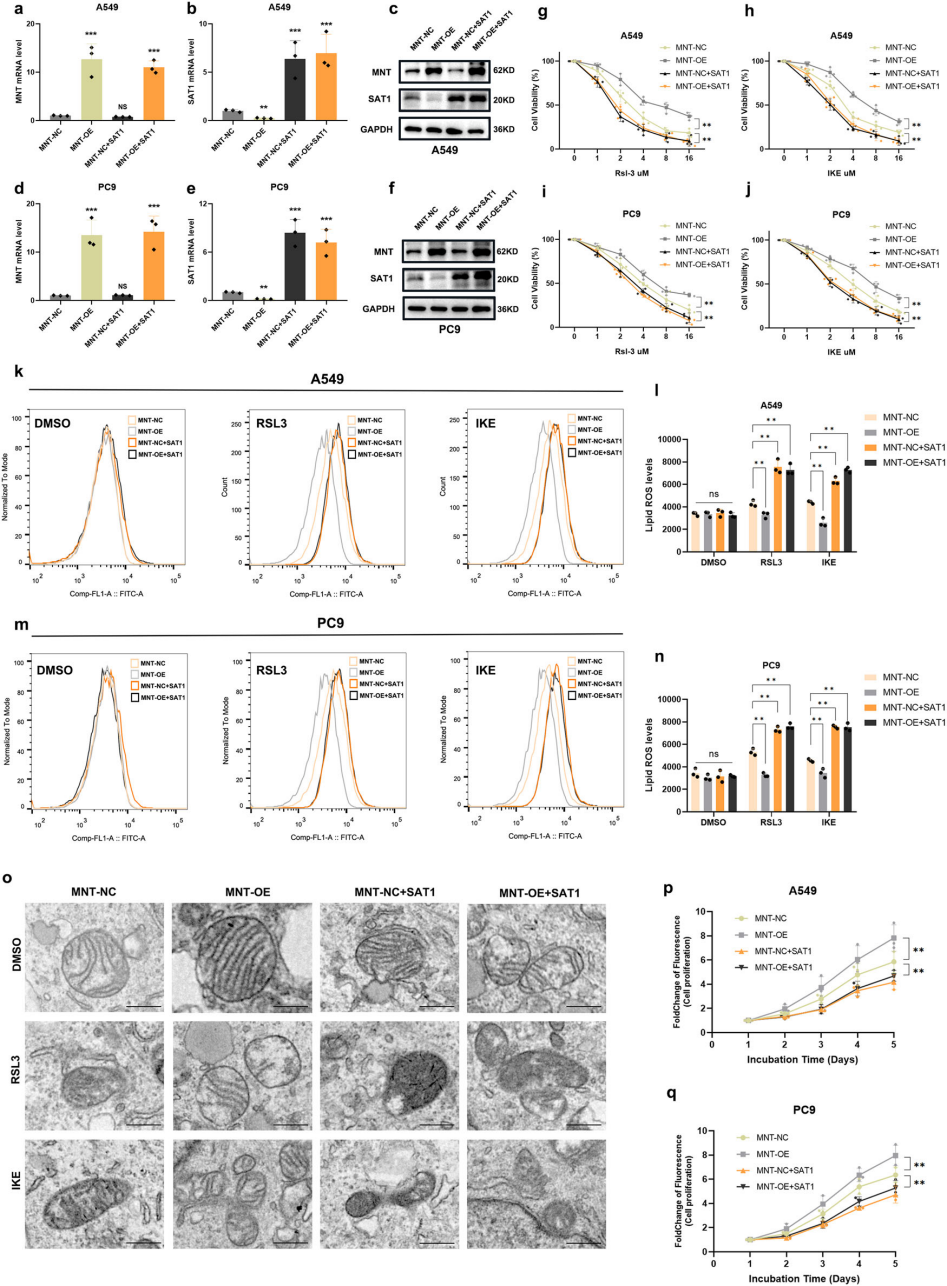
Restoring SAT1 expression antagonizes MNT’s ability to inhibit ferroptosis and promote cell proliferation. **a**–**f** qRT-PCR and western blot confirming the restored SAT1 expression in A549 and PC9 cells transfected with MNT-NC and MNT-OE. **g**–**j** Dose-toxicity curves showing the viability of A549 and PC9 cells transfected with MNT-NC, MNT-OE, MNT-NC + SAT1, and MNT-OE + SAT1 upon RSL3 or IKE treatment at the indicated concentrations for 24 h. **k**–**n** Lipid peroxidation was measured by flow cytometry after C11-BODIPY staining in MNT-NC, MNT-OE, MNT-NC + SAT1, and MNT-OE + SAT1 treatment by RSL3 or IKE (2.5 μM RSL3 and 8 μM IKE for 5 h). **o** Characteristic alterations of mitochondria in A549 cells treated with RSL3 or IKE analyzed by TEM (2.5 μM RSL3 and 8 μM IKE for 5 h) after restored SAT1 expression, Scale bars: 50 nm. **p** and **q** SAT1 expression inhibit the proliferation of A549 and PC9 cells promoted by MNT. Data were analyzed by Student’s *t*-test or two-way ANOVA and presented by mean ± SD in triplicate. ns, not significant; *p < 0.05; **p < 0.01; ***p < 0.001.

Given the above outcomes suggesting that MNT inhibits LUAD ferroptosis via suppressing SAT1 expression, we further validate by restoring SAT1 expression in the MNT-NC and OE LUAD cell lines (Fig. 5a–f). We noticed that LUAD cells became significantly more sensitive to FINs when SAT1 was upregulated (Fig. 5g–j). Conversely, the simultaneous knockdown of MNT and downregulation of SAT1 expression significantly decreased the sensitivity of LUAD cells to ferroptosis (Supplementary Fig. 3a). Additionally, after FINs treatment, MNT-NC and OE cell lines that expressed elevated SAT1 expression also had considerably higher intracellular ROS levels. (Fig. 5k–n). The TEM experiment demonstrated that cells restoring SAT1 expression displayed increased ferroptosis-sensitive characteristics with addiction to FINs, including an impaired mitochondrial morphology (Fig. 5o). Furthermore, under FINs treatment, we found that LUAD cells with restored SAT1 expression exhibited a more pronounced “Ballooning” phenotype and lower TMRM fluorescence compared to the MNT-OE and NC group (Supplementary Fig. 4a–c).

**Fig. 6:**
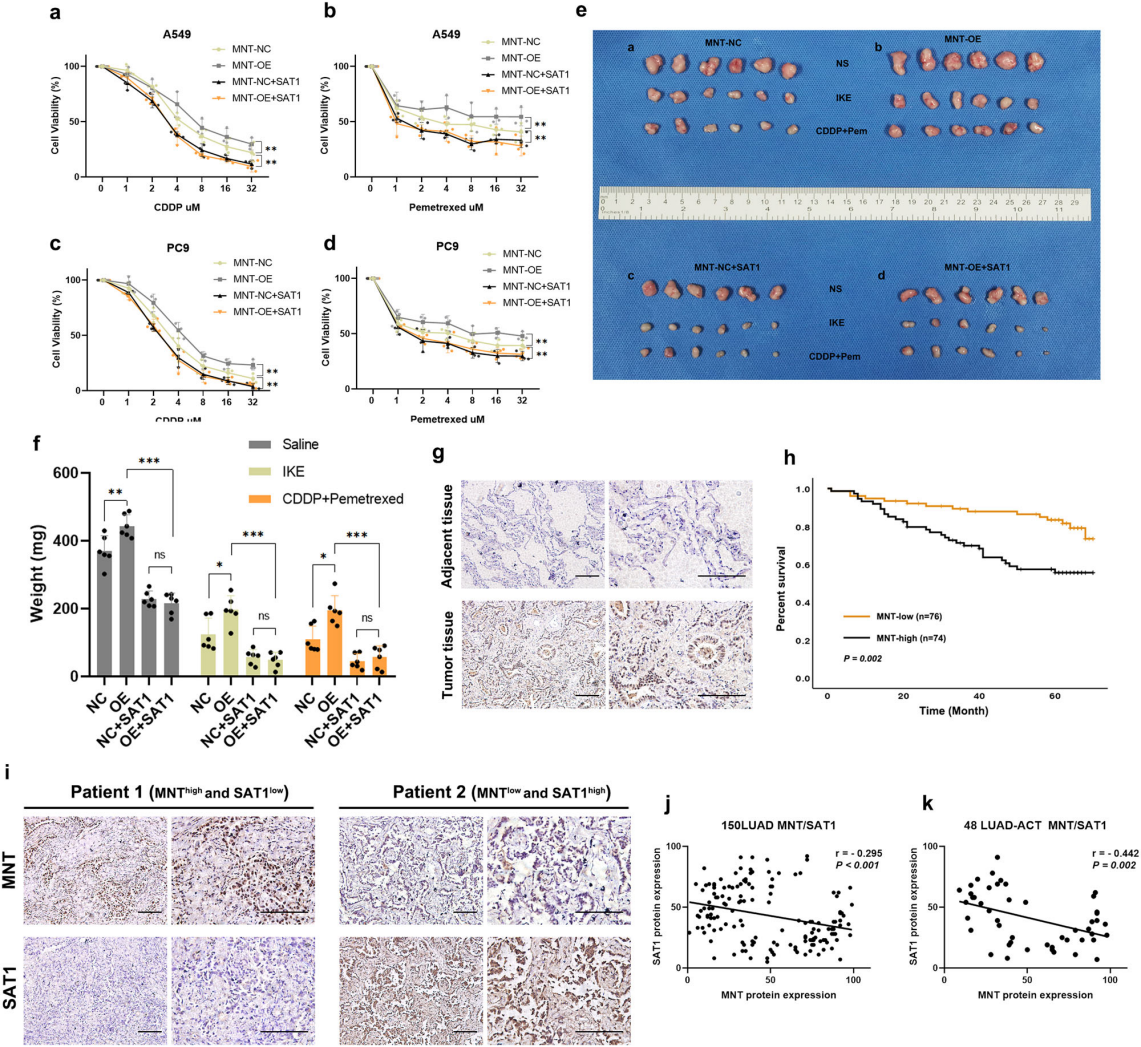
SAT1 expression antagonizes MNT’s ability to impair chemotherapy sensitivity in vitro and in vivo, MNT is highly expressed in LUAD and negatively correlates with prognosis. **a**–**d** Dose-toxicity curves showing the viability of A549 and PC9 cells transfected with MNT-NC, MNT-OE, MNT-NC + SAT1, and MNT-OE + SAT1 upon CDDP or Pem treatment at the indicated concentrations for 48 h. **e** Representative tumors formed in nude mice by MNT-NC (**a**), MNT-OE (**b**), MNT-NC + SAT1 (**c**), and MNT-OE + SAT1 (**d**) cells upon IKE or CDDP+Pem treatment. **f** The weight of the final tumor formed between different groups. **g** The expression of MNT between adjacent normal tissues and tumor tissues from patients with LUAD in our hospital. **h** High MNT expression was correlated with inferior prognosis of patients. **i**–**k** The expression of MNT was negatively correlated with SAT1. Data were analyzed by Student’s *t*-test or two-way ANOVA and presented by mean ± SD in triplicate. ns, not significant; *p < 0.05; **p < 0.01; ***p < 0.001.

In terms of the impact on proliferation, SAT1 expression dramatically reduced the capacity for proliferation in both the MNT-NC and OE cell lines (Fig. 5p, q). Fascinatingly, elevated SAT1 expression markedly increased the harmful response of MNT-inhibited chemotherapy on LUAD cells (Fig. 6a–d). The above findings imply that SAT1 can counteract the affection of MNT-mediated inhibition on ferroptosis as well as chemosensitivity and promotion of LUAD cell proliferation.

### MNT promotes tumor formation as well as suppresses ferroptosis and chemotherapy sensitivity but can be antagonized by SAT1 in vivo

We next conducted subcutaneous tumor formation by injecting deferent groups of A549 cells (MNT-NC, MNT-OE, MNT-NC + SAT1, MNT-OE + SAT1) into nude mice to validate the results obtained above in vivo (Fig. 6e). We regularly monitored tumor growth during the period, weekly measuring and documenting tumor size. The tumors were removed from the nude mice after being euthanized in 4 weeks, and the sizes were all recorded. We found the tumor size in nude mice receiving injections of MNT-OE cells was much larger in comparison to the MNT-NC group. Moreover, notable to observe is that this promotion of tumor formation was suppressed by the restoration of SAT1 expression, which is consistent with in vitro experiments (Fig. 6e, f). Additionally, various nude mouse groups were given FINs or chemotherapeutic drugs during tumor growth. It was noticed that the tumors in the MNT-NC + SAT1 and MNT-OE + SAT1 groups greatly shrank in comparison to the MNT-NC and MNT-OE groups (Fig. 6e, f). These results demonstrate that MNT increases tumor growth, suppresses ferroptosis, and decreases chemotherapy sensitivity, but that SAT1 can counteract these effects in vivo.

### MNT is highly expressed in clinical LUAD samples, associated with poor prognosis, and negatively correlated with SAT1 expression

To explore the clinical relevance of MNT in LUAD patients, we retrospectively examined the clinicopathological traits and prognosis of 150 LUAD patients with complete follow-up data in 2014 (Table S4). According to the IHC assay, we found MNT expression was noticeably low in nontumorous tissues while being substantially elevated in LUAD tissues (Fig. 6g). In addition, Kaplan–Meier analyses revealed a strong correlation between high MNT expression and inferior overall survival (OS) in LUAD patients (Fig. 6h). Furthermore, a multivariate Cox proportional hazards model indicated that high MNT expression was an independent prognostic predictor of OS (HR, 2.147, *P* = 0.025, Table S5). Out of 150 patients with LUAD, 48 patients required postoperative adjuvant chemotherapy (ACT). The clinicopathological characteristics of the 48 patients were provided in Table S6, where we classified patients as chemo-resistant and chemo-sensitive depending on whether they relapsed before the follow-up deadline. The analysis results indicated that high MNT expression was related to chemoresistance (Table S6) and that MNT expression was also inversely related to SAT1 expression (Fig. 6j, k).

## Discussion

Ferroptosis was identified in 2012 and is characterized by an iron-dependent controlled cell death pathway marked by an excess of cellular lipid peroxidation that ultimately results in plasma membrane rupture and cell death^18,19^. Emerging research underscores the pivotal role of ferroptosis as a mechanism that suppresses tumor development^7,20–22^. The present study elucidated the involvement of MNT in the regulation of ferroptosis. The findings of our study indicate that exposure to ferroptosis stress results in an upregulation of MNT expression. This upregulation of MNT was observed to suppress cellular susceptibility to both ferroptosis and chemotherapy through the inhibition of SAT1 transcription (Supplementary Fig. 3b).

SAT1, also known as Spermidine/Spermine N1-Acetyltransferase 1, is an enzyme closely associated with lipid metabolism. In some research, SAT1 has been found to regulate the process of ferroptosis. SAT1 can modulate the intracellular balance of iron ions by participating in lipid metabolism^11^, specifically in the metabolism of phosphatidylamine (PA) and phosphatidylcholine (PC), thereby affecting ferroptosis. One study confirmed that the activation of SAT1 facilitates the catabolism of spermine and spermidine, which results in the accumulation of iron and the production of lipid peroxides, subsequently inducing ferroptosis^23^. Therefore, SAT1 plays a pivotal role in the regulation of lipid metabolism and the process of ferroptosis. Upon identifying SAT1 as a downstream target of MNT, this study primarily aimed to elucidate how MNT regulates its expression. However, the regulatory mechanism of SAT1 itself on ferroptosis still requires further research for clarification.

The MNT transcription factor serves as a pivotal regulator of oncoprotein MYC, belonging to the MXD family, governing numerous cellular processes, and exhibits activation in the majority of human malignancies^14,24^. However, the functional relevance of MNT in cancer is controversial and it is unknown whether MNT is involved in ferroptosis. Our study unexpectedly found that MNT levels were significantly upregulated after the treatment of LUAD cell lines with FINs, implying that MNT may be involved in the regulation of ferroptosis in LUAD. Further experiments found different levels of MNT expression significantly affected the sensitivity of LUAD cells to FINs. In addition, MNT could promote the proliferation of LUAD cells in vitro and in vivo. To clarify the clinical relevance of MNT in patients with LUAD, we reviewed 150 lung adenocarcinoma patients with complete follow-up data and found that MNT expression was increased in LUAD cancer tissues compared with adjacent normal tissues, and that high MNT expression was associated with poor patient prognosis. These findings demonstrate that MNT plays an important role in the development and treatment of LUAD by engaging in the regulation of ferroptosis.

Structurally, as a transcription factor, MNT transcription factor has a basic helix-loop-helix-leucine zipper (bHLHLZ) structural domain and a SIN3 interacting structural domain (SID), with functions of binding to DNA and transcriptional repression, respectively^14,25^. Mechanically, MNT interacts directly with SIN3 proteins via the N-terminal SID domain, which binds to the SIN3 paired amphipathic helix (PAH2) domain of SIN3B, and then recruits histone deacetylases and other chromatin-modifying enzymes to inhibit transcription upon binding to E-boxes of target DNA^26^. We speculated that MNT might be involved in ferroptosis by regulating a key molecule in the ferroptosis pathway. To date, many key molecules play a central role in the regulatory pathways of ferroptosis, and the functions and regulatory mechanisms of some of the most well-known molecules such as GPX4, SLC7A11, and ACSL4 have been widely studied and reported^6^. in our study, however, different levels of MNT did not affect the expression of several key molecules mentioned above. We discovered that SAT1 might be a target gene of MNT using ChIP-Seq matching to the ferroptosis-related gene set. The enzyme SAT1 is responsible for regulating the initial intracellular pathway of polyamine catabolism.

Intriguingly, the upregulation of SAT1 expression triggers lipid peroxidation and enhances cellular susceptibility to ferroptosis under conditions of reactive oxygen species (ROS)-induced stress. Additionally, this process is associated with the inhibition of tumor growth in xenograft tumor models^11^. Our findings from western blot, ChIP-Seq, and dual luciferase reporter assays revealed that SAT1 is a transcriptional regulatory target molecule of MNT, which functions as an inhibitory transcription factor by interacting with SAT1’s promoter region and suppressing the gene’s transcription. We imported the promoter sequence of SAT1 into JASPAR to predict potential binding transcription factors and identified that, in addition to MNT, several other transcription factors such as SOX13, GATA1, and IKZF2 might be involved in the regulation of SAT1 (Supplementary Data 1), indicating the regulation network of SAT1 and ferroptosis is complex and needs to be further explored.

In the exploration of the clinical significance of MNT, we identified a negative association between MNT and SAT1 expression in 150 tumor tissues of LUAD patients, and the same correlation pattern was also observed in 48 tumor tissues from LUAD patients undergoing ACT. Moreover, MNT was found to increase the resistance of LUAD cells to chemotherapeutic agents in vitro and in vivo. However, this response was antagonized following the restoration of SAT1 expression. Among the 48 LUAD patients who received ACT, high MNT expression was significantly associated with chemoresistance. Numerous chemotherapeutic agents have been observed to trigger ferroptosis. Often, an imbalance in ferroptosis can result in chemotherapy resistance and subsequent treatment failure. Either pharmacological or genetic regulation of ferroptosis can effectively counteract chemotherapy resistance^27^. Since chemotherapy is still the main option for treatment for LUAD patients, the present study illustrates a potential mechanism of enhancing the sensitivity of LUAD to ferroptosis by regulating MNT, thereby overcoming chemotherapy resistance, and ultimately improving the therapeutic outcomes of chemotherapy in LUAD, .

Collectively, the data presented in this study provide evidence supporting the role of MNT as a regulator of ferroptosis, highlighting its potential therapeutic implications and clinical significance as a prognostic factor. There are some limitations in this study, such as investigating the function of MNT further at the single-cell level seems to be more insightful, as well as the fact that the use of tissue microarrays in the clinical data analysis would also be more precise.

## Materials and methods

### Cell lines

The cell lines used in this study, including A549, PC9, and HEK293T cells, were purchased and cultivated according to the procedures described in a previous study by our research group^28^. All cells were purchased from the Chinese Academy of Science Cell Bank. The cell lines were authenticated by short tandem repeat (STR) profiling in 2023 and were passaged every 3–5 days according to the cell proliferation rates. To prevent mycoplasma contamination, the cells were tested every 2 months using a PCR-based method.

### Patients and tumor specimens

This study was approved by the Ethics Committee of the Clinical Research Ethics Committee of Zhongshan Hospital, Fudan University (B2022-180R). Written informed consent was obtained from all patients. With the informed agreement, archived samples from 150 LUAD patients of our hospital in 2014 were used for clinicopathologic analysis as demonstrated in the previous study^28^. The TNM classification, according to the AJCC Cancer Staging Manual, Eighth Edition, was utilized to determine the tumor stage. The WHO guidelines served as the basis for the pathologic classification. November 2019 was defined as the end of the follow-up procedure. Overall survival (OS) was established as the duration of the surgical procedure until either the occurrence of mortality or the most recent recorded follow-up for patients who remained alive. All ethical regulations relevant to human research participants were followed

### Compounds

The following compounds were supplied by Topscience (Shanghai, China): RSL3 (T3646), IKE (T1765), ferrostatin-1 (T6500), deferoxamine (DFO; T1637), CisDiamminedichloride platinum (CDDP; T1564), and Pemetrexed (Pem; T6226). The initial four reagents were solved in DMSO, while the latter two were dissolved in PBS. The resulting solutions were subsequently preserved at a temperature of −80 °C.

### Quantitative real-time PCR

Extraction of RNA and qRT-PCR was carried out following methods previously described^29^ In brief, The TRIzol compound (TIANGEN) was employed for the extraction of total RNA from cells, and a Hifair II First-Strand cDNA Synthesis Kit (gDNA Digester Plus, YEASEN, China) was used to synthesize cDNA. The ABI QuantStudio 5 real-time PCR machine (Thermo Fisher, USA) was used to execute a Hifair III One-Step qRT-PCR reaction. Quantitative analysis was performed using the 2^(-△△CT) technique as described previously^28^. Table S1 contains a complete listing of the primers that were generated for each gene by Sangon Biotech.

### Cell viability assays

In the context of cytotoxicity assays, a total of 3000 cells were allocated in 96-well plates along with subsequently subjected to a 24 h incubation period. The cells were subjected to treatment with various concentrations of reagents for a duration of 48 h, according to per the experimental protocol. In the context of proliferation experiments, cell lines were initially implanted on an amount of 1000 each well and subsequently subjected to incubation for 0, 24, 48, 72, 96, and 120 h at a temperature of 37 °C. The assessment of cellular viability was conducted using CCK-8 (Beyotime) following guidelines provided by the manufacturer.

### Chromatin immunoprecipitation (ChIP) assays

The SimpleChIP® Plus Enzymatic Chromatin IP Kit (Magnetic Beads) (CST) was utilized for the execution of the ChIP assay. Initially, the cells underwent cross-linking, followed by membrane lysis and treatment with micrococcal nuclease to fragment chromatin. Subsequently, the chromatin that had been cross-linked and digested was subjected to incubation with Flag-Tag Antibody (CST). The positive control in this study was the Histone H3 antibody obtained from CST, while the negative control was the human IgG also obtained from CST. Table S2 provides a comprehensive listing of antibodies. Following a comprehensive washing process, the DNA was extracted from the beads and subsequently utilized in qRT-PCR.

### Lentiviral infections

The lentivirus vectors MNT and SAT1, along with their respective negative control sequences, were procured from Genechem Technology to establish cells with stable overexpression of MNT or SAT1. Furthermore, the viral vector utilized for knock-down MNT expression, along with the negative control sequences, were procured from Genechem Technology (as indicated in Table S3). The methodology employed for the summary involves the seeding of a combined number of 5 × 10^4^ cells into 12-well plates. Twenty-four hours latter, lentivirus was introduced at a multiplicity of infection (MOI) of 10. Then, cell lines were cultured in a complete medium supplemented with 5 mg/mL polybrene for 12 h. Following this, fresh medium was used to replace the culture medium. Ultimately, the cells were cultured in the medium supplemented with 2.5 mM puromycin after 72 h transduction.

### Lipid peroxidation detection

The experiment involved seeding cells in 12-well plates and administering compounds the following day. Briefly, the cells were incubated with 1 ml of fresh medium containing 5 μM of BODIPY 581/591 C11 (Invitrogen) for 30 min^30^. Subsequently, the cells underwent trypsinization, followed by washing and resuspension in 500ul of PBS preparing for flow cytometry assay, the gating strategies were presented in Supplementary Fig. 6. A total of at least 20,000 cells were examined for each experimental condition. The data was assessed utilizing the FlowJo software (TreeStar, Woodburn, OR, USA).

### Western blotting

In brief, cellular lysis was performed utilizing RIPA buffer (Beyotime) supplementary with a cocktail of protease and phosphatase inhibitors. The proteins acquired were quantified using an Enhanced BCA Protein Assay Kit (Beyotime) and subsequently subjected to boiling in 5 × SDS-PAGE loading buffer (EpiZyme, Shanghai, China) for 10 min at a temperature of 100 °C. Subsequently, the proteins underwent SDS-PAGE in preparation for western blotting. The antibodies employed in this study were as follows: MNT (1:1000, #DF4676), SAT1(1:1000, #DF12469), GPX4 (1:1000, #DF6701), and SLC7A11 (1:1000, #DF12509) from Affinity Biosciences, ASCL4 (1:1000, #abs106075) from Absin, and GAPDH (1:3000, # AG019) from Beyotime. All original strips of the western blotting in this study were presented in Supplementary Fig. 5

### Dual-luciferase reporter assays

This transcript (−1000 ~ 200 bp around the Transcription Starting Site) was cloned into the phy-811@7 dual luciferase reporter vector (Hanyin Technology, Shanghai, China) of the SAT1 promoter region. At 60 %–70 % confluence, HEK293T cells were planted in a 24-well plate that had been pre-treated with polylysine. After 24 h, the cells were co-transfected with Lipo8000 (Beyotime) and 200 Nm MNT mimics together with 400 ng of the wild-type or mutant plasmids. After 48 h, cells were collected and analyzed utilizing a Luciferase Reporter Gene Assay Kit (Beyotime) to perform dual-luciferase reporter experiments. Finally, luciferase activity was evaluated by Microplate spectrophotometer (Bio-Rad, Hercules, CA, USA).

### Immunohistochemistry (IHC)

Immunohistochemical staining was performed using MNT antibody (1:100, Affinity, # DF4676), SAT1 antibody (1:100, Affinity, #DF12469), and GTVisionTM III Detection System (Gene Tech, Shanghai, China). The quantification of positive staining intensity was conducted following the previously outlined methodology^31^. Subsequently, the expression of the target was dichotomized into high and low categories, utilizing a predetermined threshold of 40 %.

### Transmission electron microscopy

A 2.5 % glutaraldehyde solution was used to fix cells grown in 6 cm dishes. Cells were prefixed with phosphate buffer containing 1 % osmic acid, washed three times in 0.1 M PBS. After dehydrating and embedding the cells, they spent 48 h in an oven heated to 60°C. Lead citrate and uranyl acetate were used to stain ultrathin sections. The next day, we used a Hitachi transmission electron microscope (Hitachi, Japan) to examine the dried sections.

### Tumor xenograft model in Nude mice

Animal research was approved by the Animal Ethics Committee of Zhongshan Hospital and the experiments were conducted in accordance with the guidelines. We have complied with all relevant ethical regulations for animal use. A total of six million A549 cells that had undergone a variety of interventions were suspended in 300 μl of culture medium and then injected subcutaneously into the left side of 4 week-old male BALB/c nude mice. The tumors were collected at the end of 4 weeks following implantation. The tumors’ weights were measured and a calculation was used to determine their volumes (length × width^2^)/2.

### Statistics and reproducibility

R software, GraphPad Prism version 8.0, and SPSS 25.0 gram (IBM SPSS, Chicago, IL, USA) were used to conduct the statistical analyses. Unpaired Student’s *t*-tests and the Wilcoxon test were used in the statistical analysis for comparing continuous variables between two different groups. Spearman coefficient tests were utilized to conduct a correlation analysis between MNT and SAT1. The statistical technique of Kaplan–Meier was employed to conduct survival analysis, along with the log-rank test. The study employed a Cox proportional hazards regression model to examine the independent prognostic factors. The statistical outcomes are exhibited in the form of means, and the vertical lines extending from the means indicate the standard deviation unless explicitly mentioned otherwise. All *p*-values were two-tailed, and a significance level of *p* < 0.05 was deemed appropriate. The notation used to indicate statistical significance was as follows: *p < 0.05, **p < 0.01, ***p < 0.001, and ns was used to indicate non-significance. The study’s experimental procedures were replicated thrice.

### Reporting summary

Further information on research design is available in the Nature Portfolio Reporting Summary linked to this article.

### Supplementary information

Supplementary Information

Supplementary Data 1

Supplementary Data 2

Description of Supplementary Materials

Reporting Summary

## Data Availability

RNA-Seq data including differential gene expression table and clinical data for the 150 LUAD patients have been deposited in Figshare (10.6084/m9.figshare.24847179, 10.6084/m9.figshare.24847218, 10.6084/m9.figshare.24847221)^32–34^. Transcription factors predicted to potentially bind to the SAT1 promoter region are presented in Supplementary Data 1, and the numerical source data were presented in Supplementary Data 2. Original Western blots are presented in Supplementary Fig. 5. Other source data supporting the findings of this study are available from the corresponding author upon reasonable request.
