# Supplementary material for: MNT inhibits lung adenocarcinoma ferroptosis and chemosensitivity by suppressing SAT1
Source: Commun Biol. 2024 Jun 3;7:680. doi: 10.1038/s42003-024-06373-5 (PMC11148173; doi:10.1038/s42003-024-06373-5)
Supplement: Supplementary file 1 — Supplementary Information [file 42003_2024_6373_MOESM1_ESM.pdf]

Supplementary Figure 1

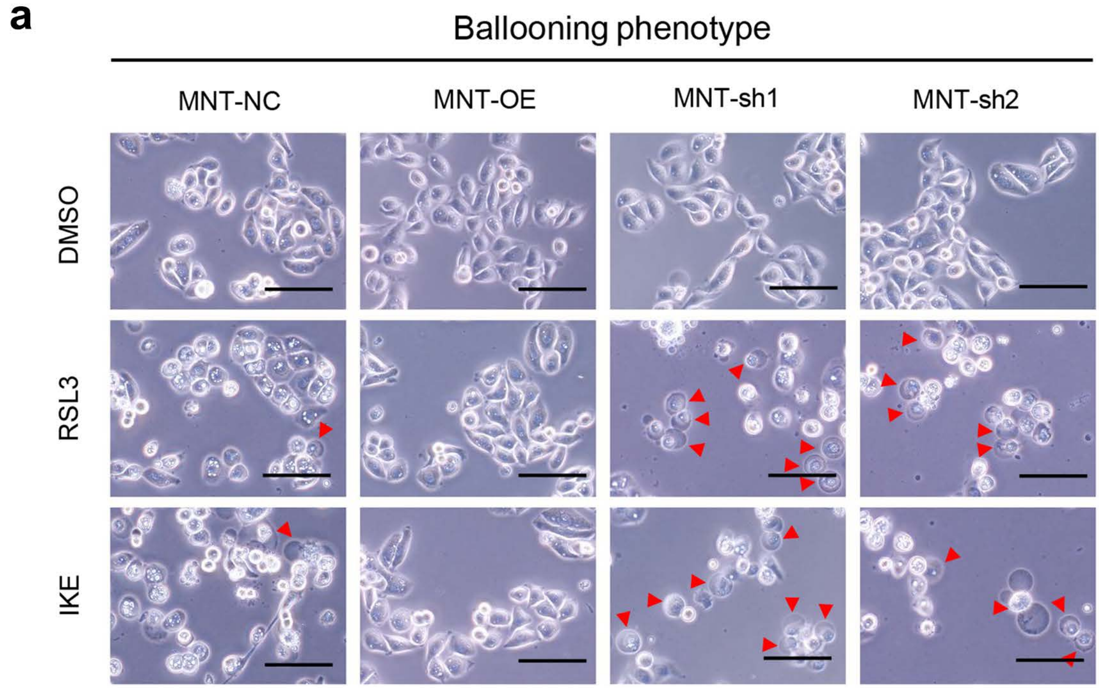

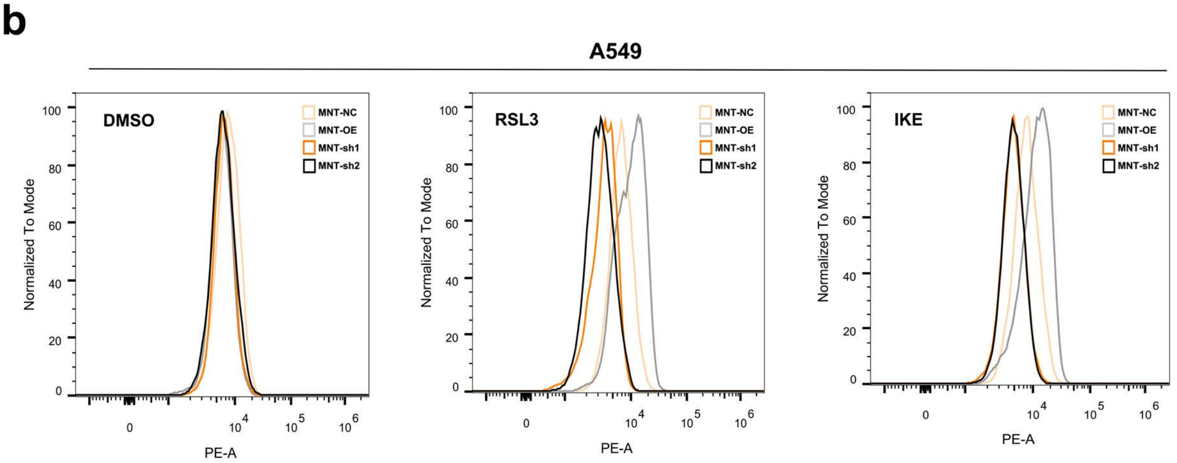

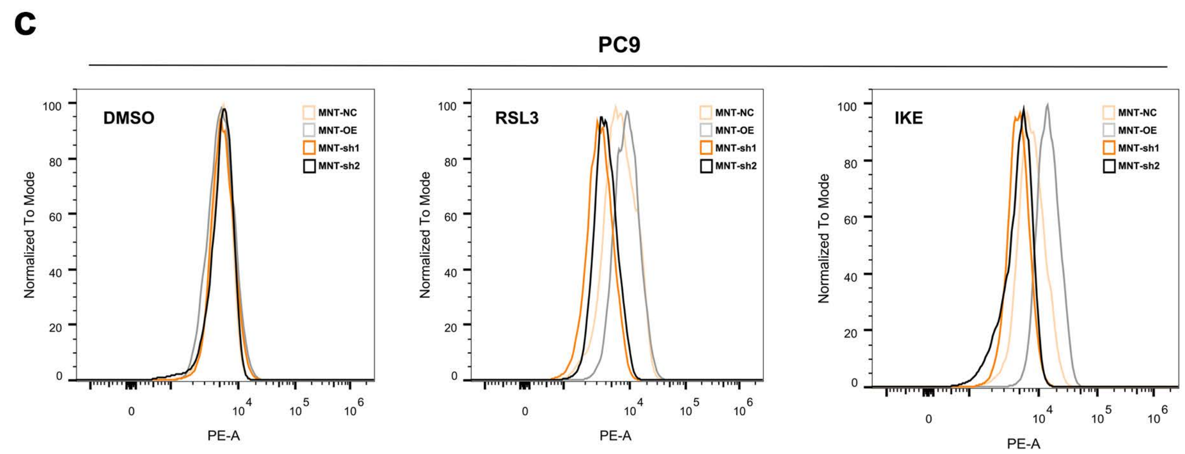

Supplementary Figure 2

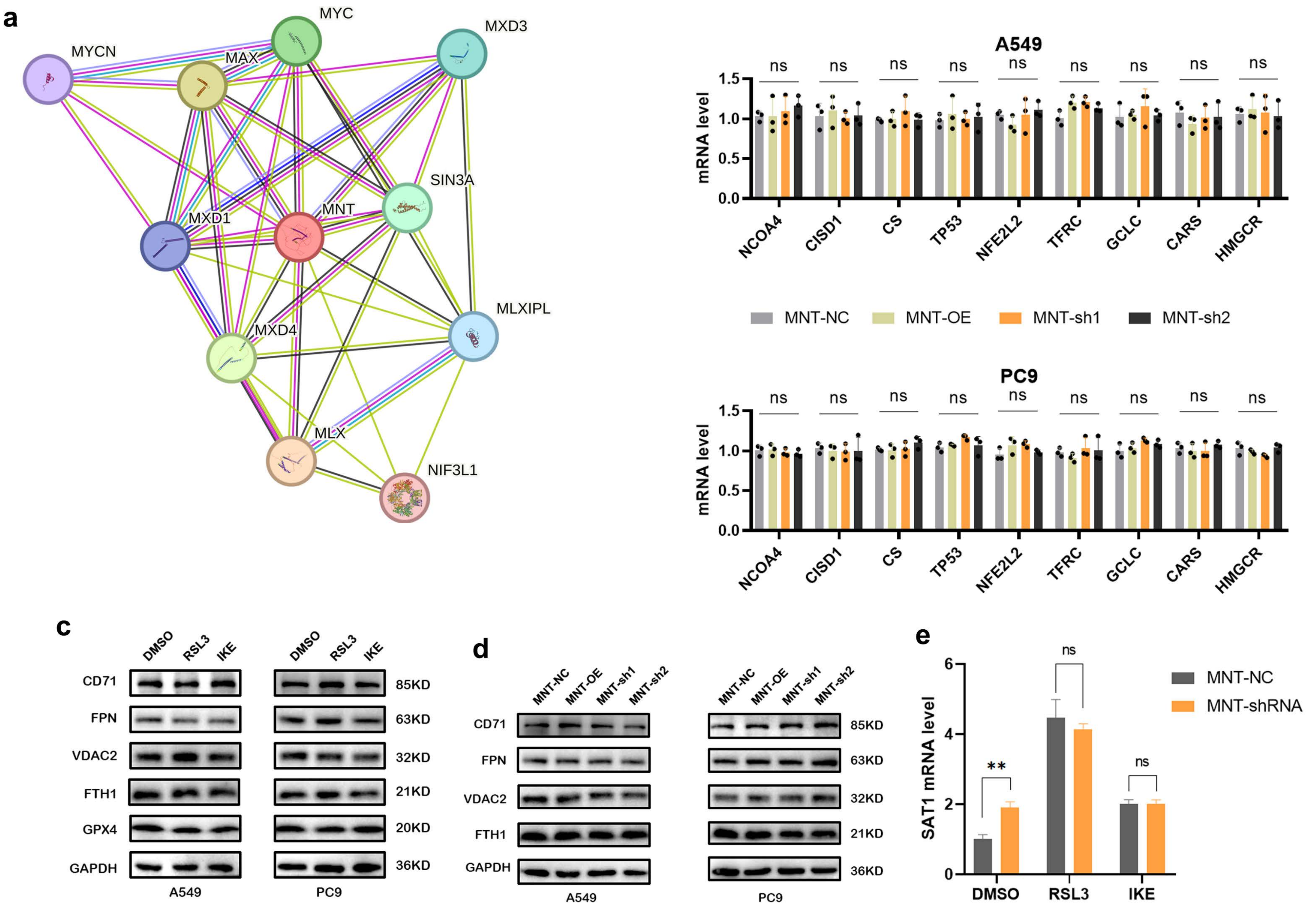

Supplementary Figure 3

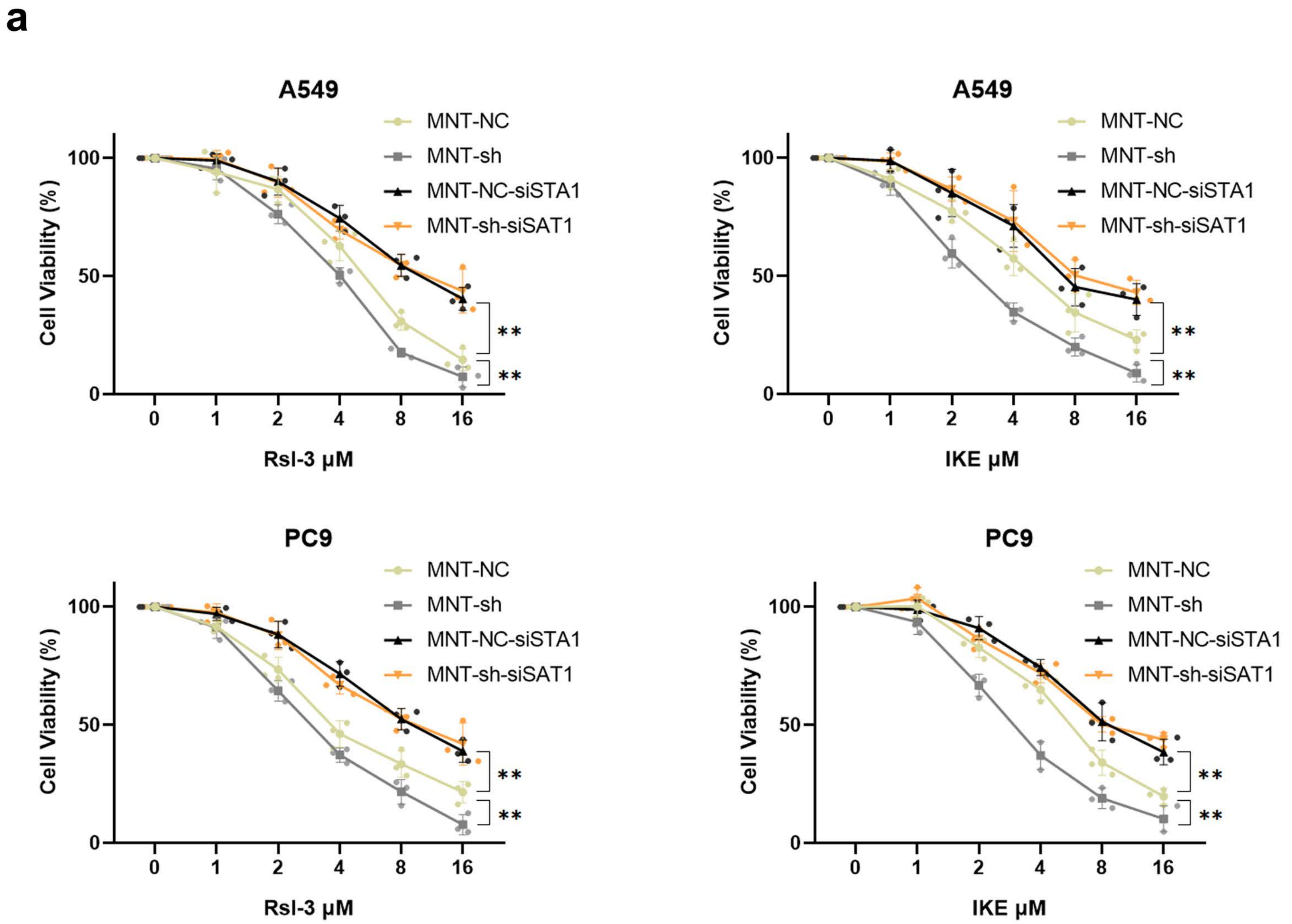

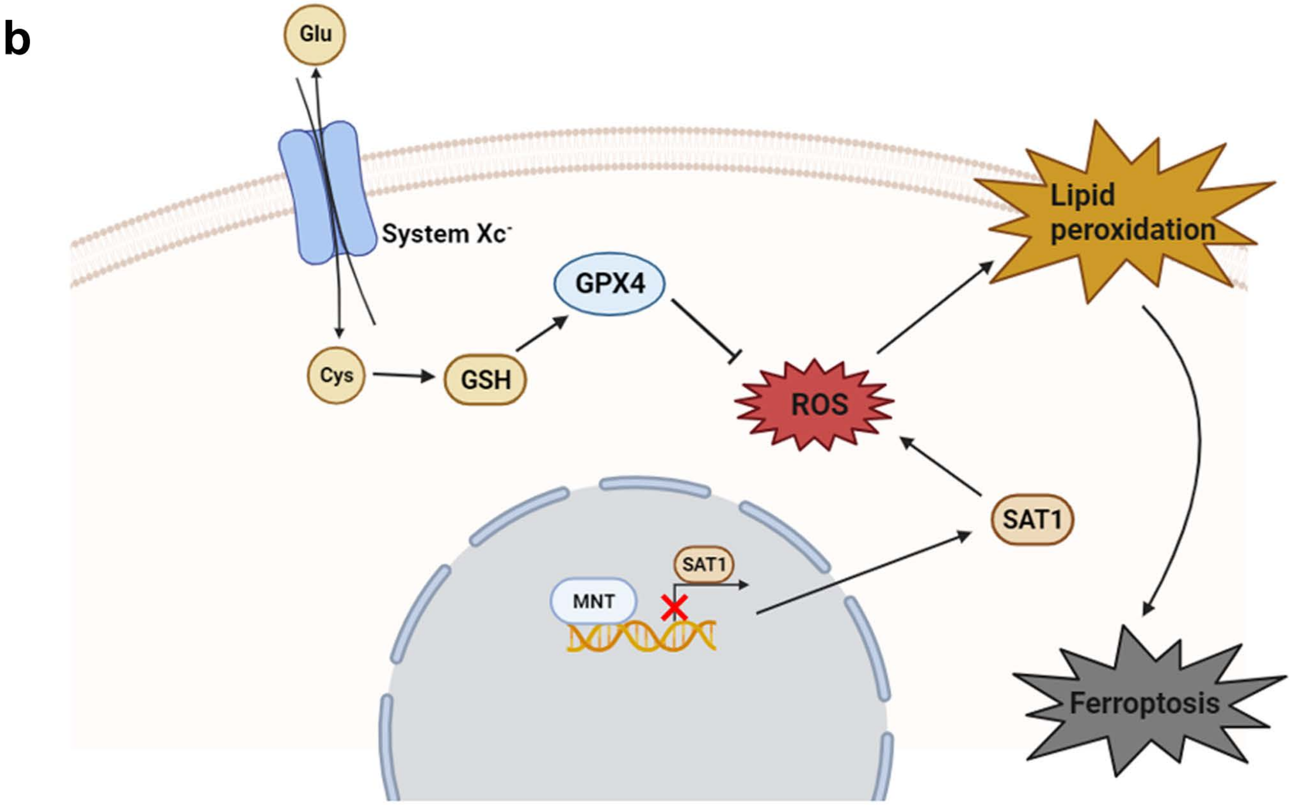

Supplementary Figure 4

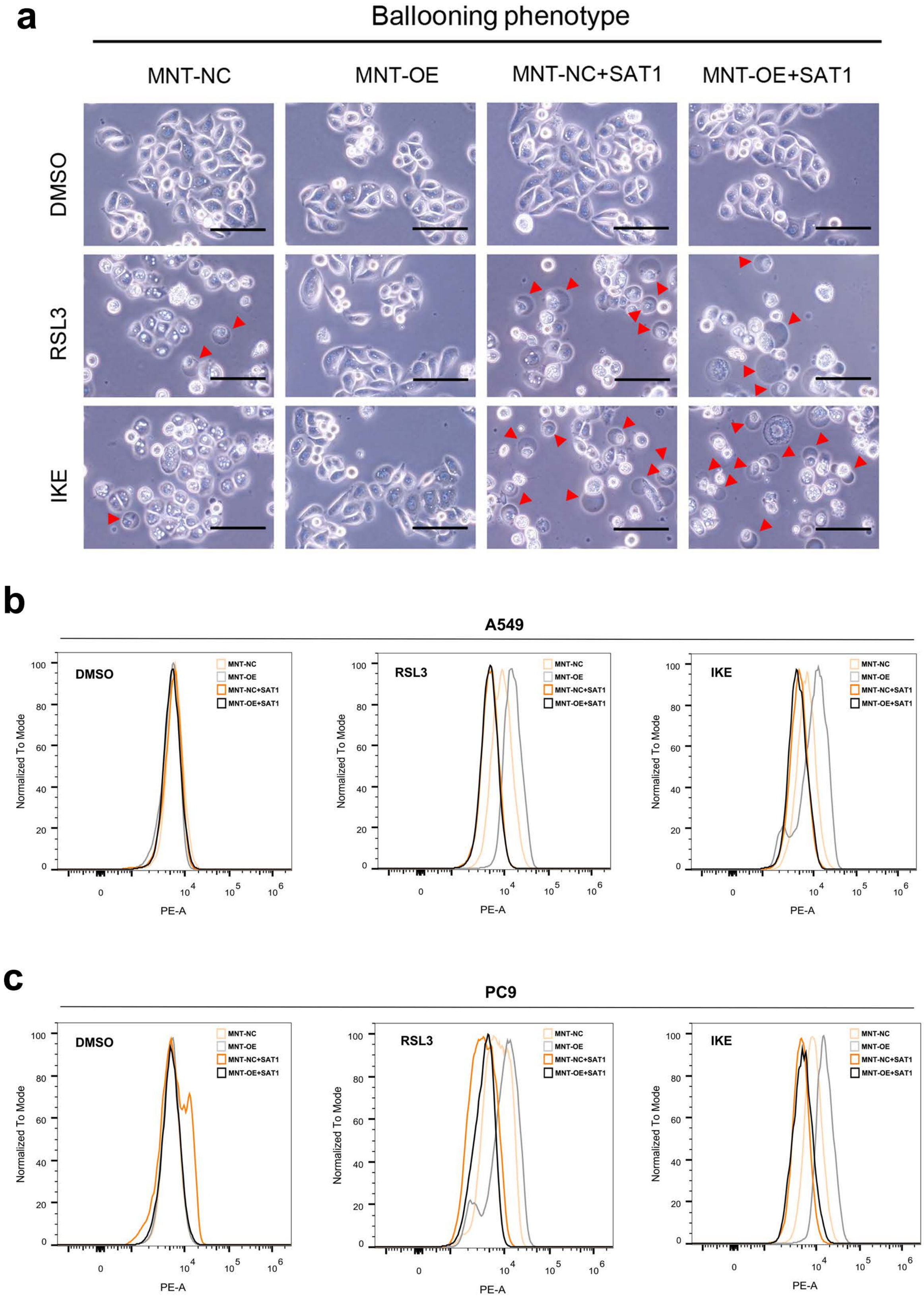

Supplementary Figure 5

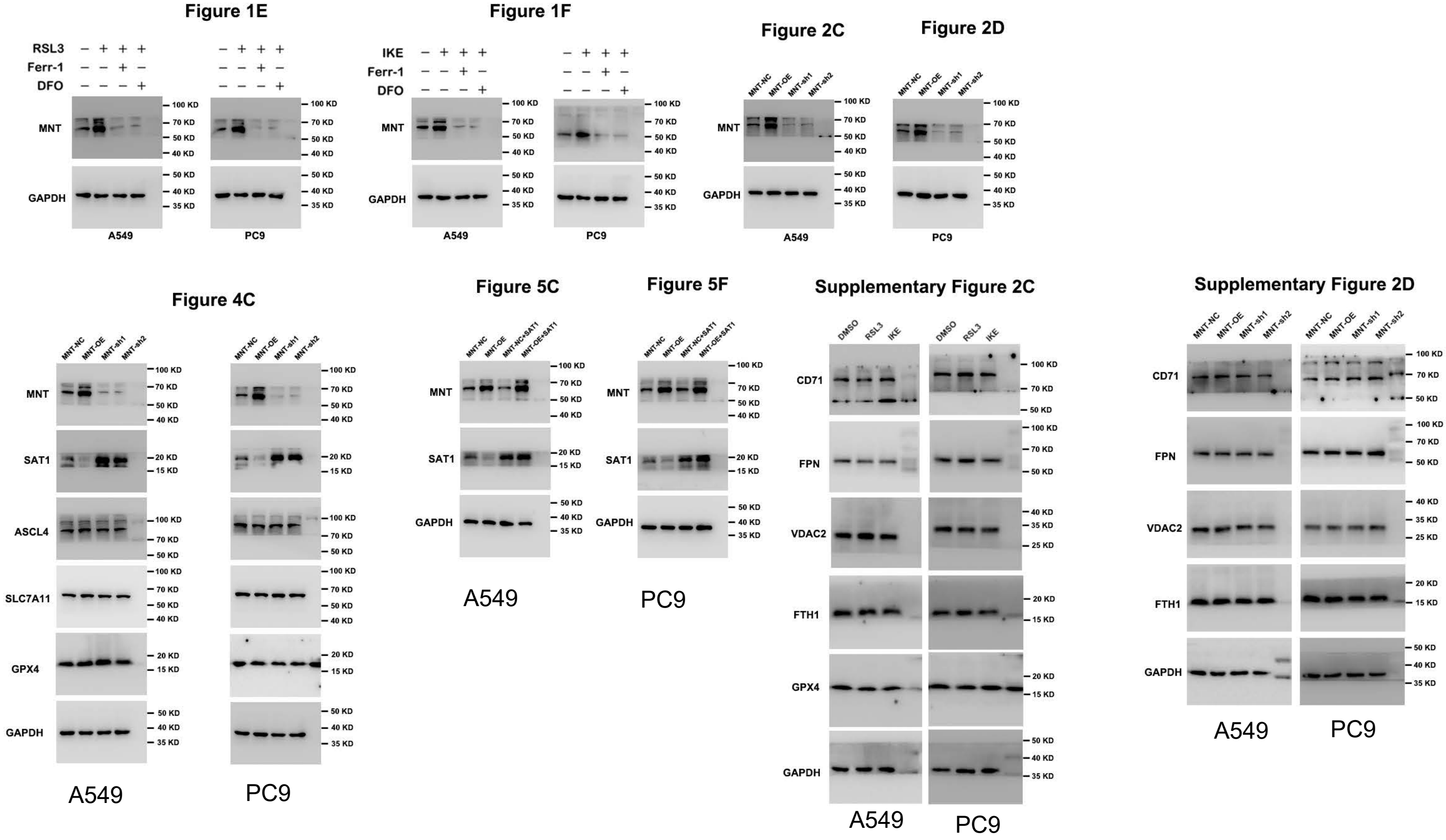

Supplementary Figure 6

Figure 2i and j

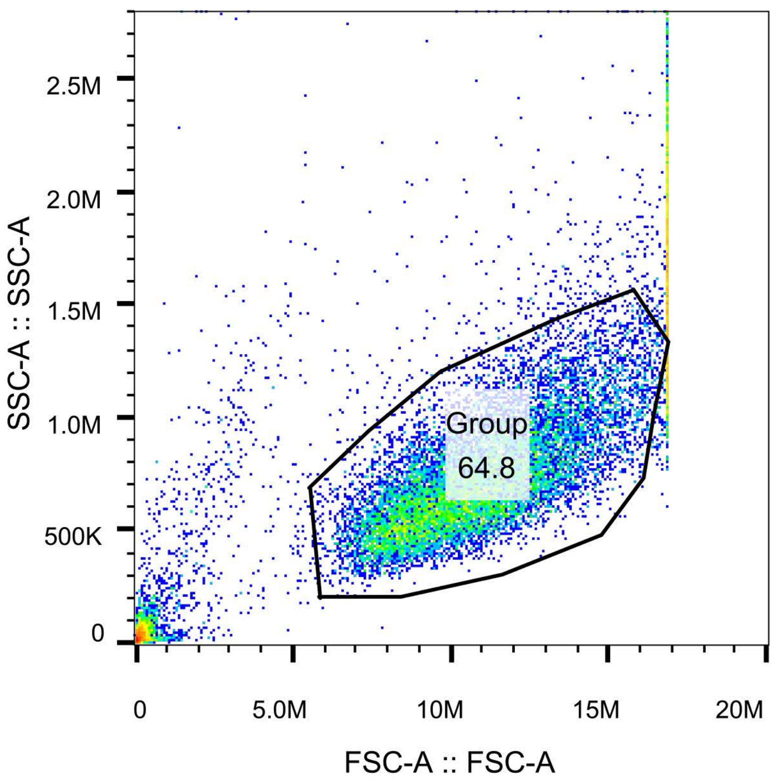

Figure 5k and m

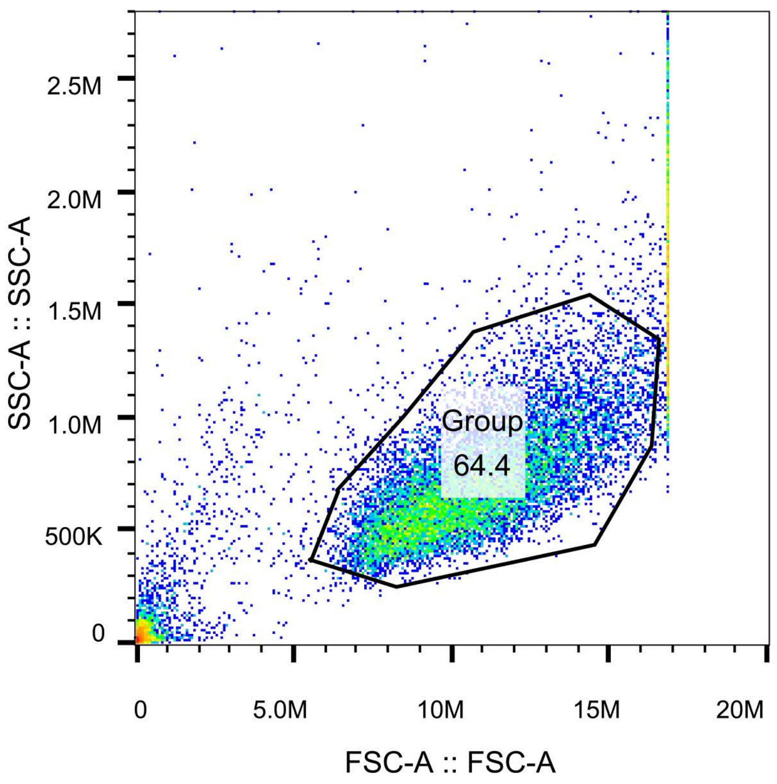

Supplementary Figure 1b and c

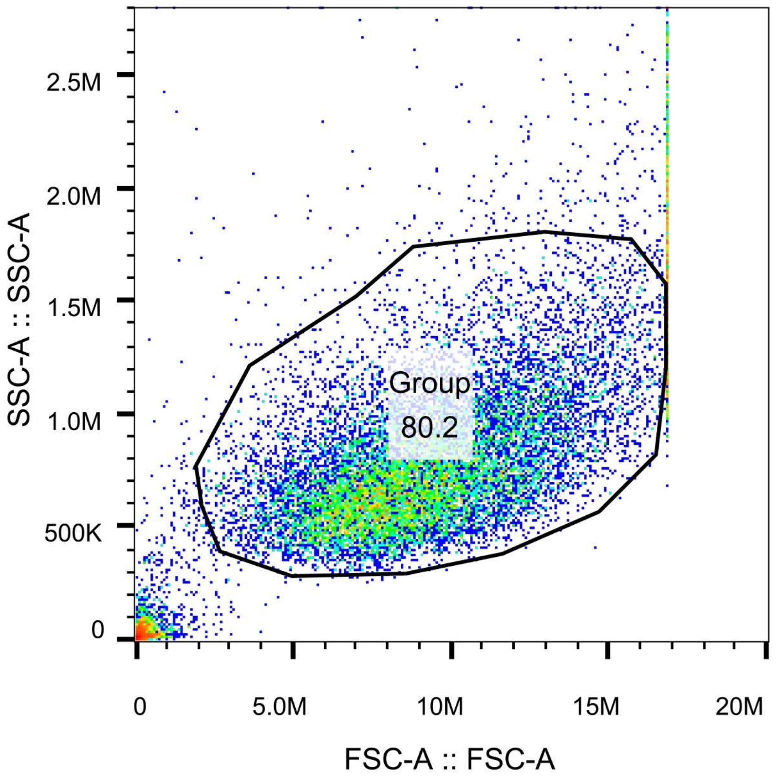

Supplementary Figure 4b and c

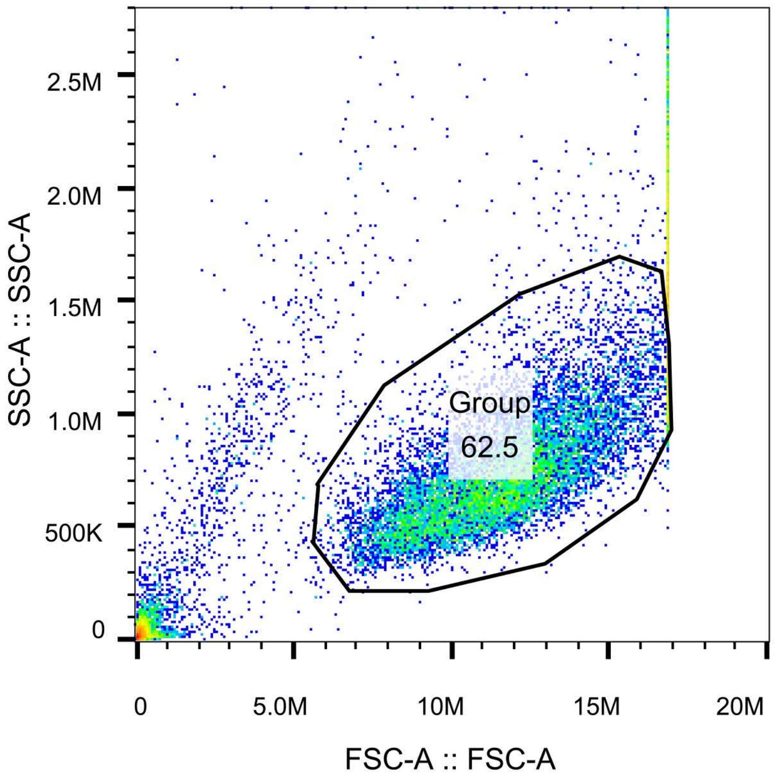

Supplementary Figure legends

Supplementary Figure 1. (a) Optical microscopy images illustrate the presence or absence of the ballooning phenotype in MNT-NC, MNT-OE, and MNT-sh cells treatment by RSL3 or IKE (2.5μM RSL3 and 8μM IKE for 5 hours), with red arrows indicating typical ballooning morphology, Scale bars 50 μm. (b and c) Mitochondrial membrane potential was analyzed using TMRM fluorescence detected by flow cytometry in MNT-NC, MNT-OE, and MNT-sh cells treatment by RSL3 or IKE.

Supplementary Figure 2. (a) Retrieved partners binding with MNT through the String database. (b) qPCR results showed differences in RNA levels of the key molecules of ferroptosis between the MNT-NC, MNT-OE, and MNT-sh of A549 and PC9 cell lines. (c and d) Western blot results showed differences in protein levels of the key molecules of ferroptosis upon the treatment of FINs in LUAD cells and the MNT-NC, MNT-OE, and MNT-sh of LUAD cell lines. (e) qPCR assessed SAT1 expression changes in FINs-treated, MNT-knockdown A549 cells

Supplementary Figure 3. (a) Dose-toxicity curves showing the viability of A549 and PC9 cells transfected with MNT-NC, MNT-sh, MNT-NC-siSAT1, and MNT-sh-siSAT1 upon RSL3 or IKE treatment at the indicated concentrations for 24h. (b) Mechanism of MNT regulation of SAT1 transcription.

Supplementary Figure 4. (a) Optical microscopy images illustrate the presence or absence of the ballooning phenotype in MNT-NC, MNT-OE, MNT-NC+SAT1, and MNT-OE+SAT1 cells treatment by RSL3 or IKE (2.5μM RSL3 and 8μM IKE for 5 hours), with red arrows indicating

typical ballooning morphology, Scale bars 50 µm. (b and c) Mitochondrial membrane potential was analyzed using TMRM fluorescence detected by flow cytometry in MNT-NC, MNT-OE, MNT-NC+SAT1, and MNT-OE+SAT1 cells treatment by RSL3 or IKE.

Supplementary Figure 5. All original strips of the western blotting in this study.

Supplementary Figure 6. The gating strategies of flow cytometry in this study.

Table S1. Sequences of Primers for Quantitative real-time-Polymerase Chain Reaction Analysis

Gene	Forward Primer: 5'-3'	Reverse Primer: 5'-3'
MNT	TCGTGTGCTGCTGCTGCTATTG	ACTCCAGACCAGCCACTTCCTATG
GPX4	AGAGATCAAAGAGTTCGCCGC	TCTTCATCCACTTCCACAGCG
SAT1	ATGACCCGTGGATTGGCAAGTT	ACAGCAGCACTCCTCACTCCT
SLC7A11	GGTGGAACGAGGAGGTGGAGAA	TTGGAGATGGTGGACACAACAGG
ASCL4	ATGTCTGCTTCTGCTGCCCAAT	AACCGCCTTCTTGCCAGTCTT

Table S2. Details of antibodies used in this research

Protein	Supplier	Cat No.	Source	Application	Concentration
ACSL4	Absin	abs106075	Rabbit	western blot	1:1000
MNT	Affinity	DF4676	Rabbit	western blot, IHC	1:1000, 1:50
SAT1	Affinity	DF12469	Rabbit	western blot, IHC	1:1000, 1:50
Flag-Tag	CST	14793S	Rabbit	ChIP	1:50
GPX4	Affinity	DF6701	Rabbit	western blot, IHC	1:1000, 1:100
SLC7A11	Affinity	DF12509	Rabbit	western blot	1:1000
GAPDH	Beyotime	AG019	Mouse	western blot	1:3000

Table S3. Sequences of shRNA

Name	Sequence (5'-3')
MNT-shRNA-1	GGATGGACGTACTGGAGAT
MNT-shRNA-2	GCACCAGCTGAAGAAGTCA

Table S4. Clinicopathologic Variables in 150 Patients With LUAD

**Variable**	**No. of Patients**	**MNT expression**		***P***
		**Low**	**High**	
Age				
≤62	80	45	35	0.070
> 62	70	29	41	
Sex				
Male	67	29	38	0.183
Female	83	45	38	
Smoking status				
Yes	29	8	21	0.009
No	121	66	55	
Stage				
I-II	120	66	54	0.005
III-IV	30	8	22	
Lymph node metastasis				
Yes	42	11	31	<0.001
No	108	63	45	
Tumor size				
≤3cm	66	38	28	0.073
> 3cm	84	36	48	
SAT1 expression				
Low	77	22	55	<0.001
High	73	52	21	

Table S5 Univariate and Multivariate Analyses of Factors Associated With Overall Survival

Variables	Univariate analyse HR (95% CI)	*P*	Multivariate analyse HR (95% CI)	*P*
Age (> 62 vs ≤ 62)	1.261(0.706-2.252)	0.434		
Gender (Male vs Female)	2.080(1.155-3.746)	0.015	1.777(0.970-3.254)	0.063
Smoking (Smoker vs Non-smoker)	1.888(0.973-3.663)	0.060		
Tumor size (>=3cm vs <3cm)	5.298(2.368-11.854)	<0.001	2.944(1.216-7.128)	0.017
Lymph node- metastasis (Yes vs No)	6.527(3.536-12.045)	<0.001	3.450(1.740-6.840)	<0.001
Tumor stage (III-IV vs I-II)	3.763(2.092-6.771)	<0.001	0.614(0.281-1.345)	0.223
MNT expression (High vs Low)	3.104(1.629-5.915)	0.001	2.147(1.103-4.178)	0.025

Abbreviations: CI, confidence interval; HR, hazard ratio; OS, overall survival.

A Cox proportional hazards regression model was used for multivariate analysis.

Table S6. Clinicopathological characteristics of 48 LUAD patients undergoing postoperative adjuvant chemotherapy(ACT).

**Variable**	**No. of Patients**	**ACT**		***P***
		**Sensitive**	**Resistant**	
Age				
≤62	22	10	12	0.827
> 62	26	11	15	
Sex				
Male	23	8	15	0.230
Female	25	13	12	
Smoking status				
Yes	9	3	6	0.712
No	39	18	21	
Stage				
I-II	37	21	16	0.001
III-IV	11	0	11	
Lymph node metastasis				
Yes	15	1	14	< 0.001
No	33	20	13	
Tumor size				
≤3cm	16	11	10	0.029
> 3cm	32	5	22	
MNT expression				
Low	24	15	9	0.009
High	24	6	18	
