# Supplementary material for: MNT inhibits lung adenocarcinoma ferroptosis and chemosensitivity by suppressing SAT1
Source: Commun Biol. 2024 Jun 3;7:680. doi: 10.1038/s42003-024-06373-5 (PMC11148173; doi:10.1038/s42003-024-06373-5)
Supplement: Supplementary file 4 — Description of Supplementary Materials [file 42003_2024_6373_MOESM4_ESM.docx]

**Description of Additional Supplementary Files**

**File name:** Supplementary Data 1

**Description:** Predictive Results

**File name:** Supplementary Data 2

**Description:** Numerical source data
